# Supplementary material for: Global Distribution Patterns and Climatic Drivers of Plant Diversity in Rubiaceae
Source: Biology (Basel). 2025 Dec 1;14(12):1719. doi: 10.3390/biology14121719 (PMC12730950; doi:10.3390/biology14121719)

Supplementary Material

Fig. S1 Distribution pattern of species density in the Rubiaceae family. (a) Overall distribution of species density in the Rubiaceae family (SD_{total}). (b) Distribution pattern of species density in the herbaceous growth type Rubiaceae family (SD_{herb}). (c) Distribution pattern of species density in the woody growth type Rubiaceae family (SD_{woody})

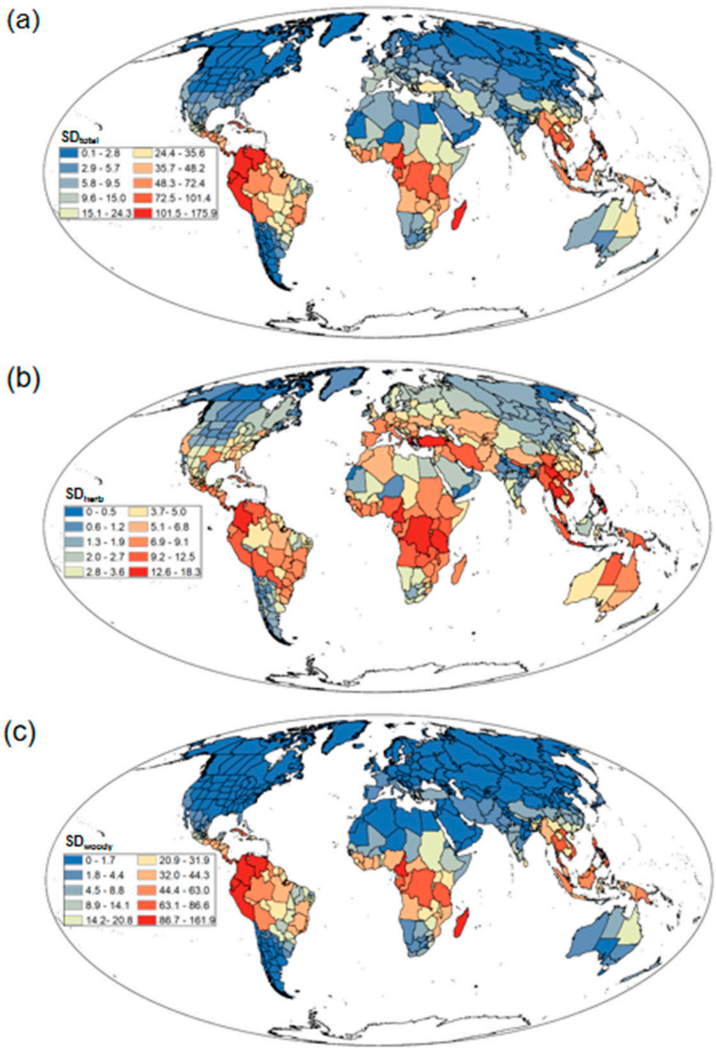

Fig. S2 Standardized regression coefficients between different species density components and sevenenvironmental variables based on SAR analysis. (a) Total Rubiaceae species density (SDtotal); (b) herbaceous Rubiaceae species density (SDherb); (c) woody Rubiaceae species density (SDwoody); (d) the ratio of herbaceous Rubiaceae species density to total Rubiaceae species density (Ratio). The blue line represents water and energy, the green line represents climate seasonality, the red line represents historical climate change, and the yellow line represents human influence. The environmental factors on the Y-axis include Annual Mean Temperature (AMT), Actual Evapotranspiration (AET), Temperature Seasonality (log-transformed) (TS-log), Precipitation Seasonality (log-transformed) (PS-log), AMT anomaly (AMTano), AP anomaly (APano), and Human Influence Index (HII), with * indicating significant effects.

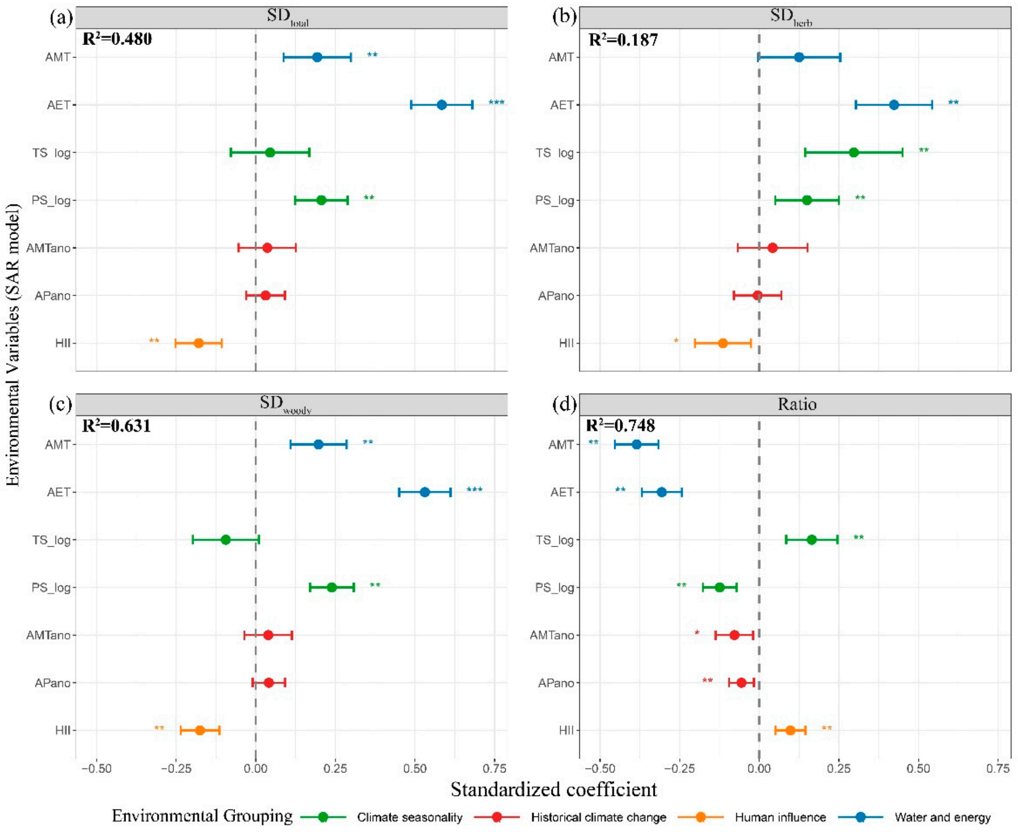

Fig. S3 Standardized regression coefficients between different species richness components and seven environmental variables based on OLS analysis. (a) Total Rubiaceae species density (SRtotal); (b) herbaceous Rubiaceae species richness (SRherb); (c) woody Rubiaceae species richness (SRwoody); (d) the ratio of herbaceous Rubiaceae species richness to total Rubiaceae species richness (Ratio). The blue line represents water and energy, the green line represents climate seasonality, the red line represents historical climate change, and the yellow line represents human influence. The environmental factors on the Y-axis include Annual Mean Temperature (AMT), Actual Evapotranspiration (AET), Temperature Seasonality (log-transformed) (TS-log), Precipitation Seasonality (log-transformed) (PS-log), AMT anomaly (AMTano), AP anomaly (APano), and Human Influence Index (HII), with * indicating significant effects.

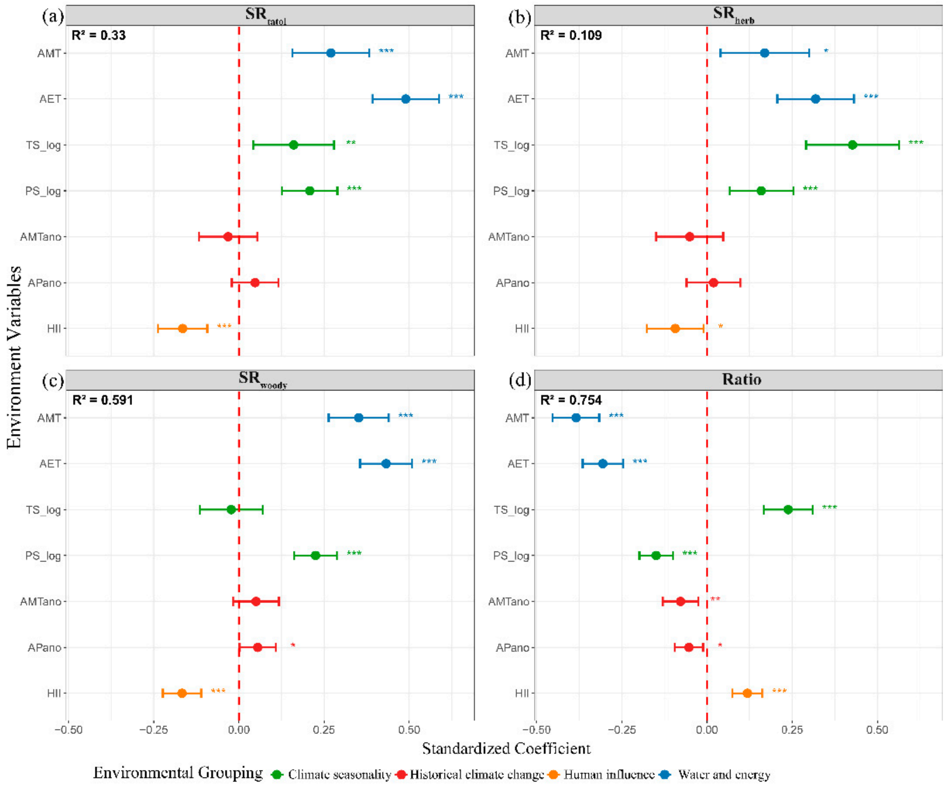

Fig. S4 Standardized regression coefficients between different species density components and sevenenvironmental variables based on OLS analysis. (a) Total Rubiaceae species density (SDtotal); (b) herbaceous Rubiaceae species density (SDherb); (c) woody Rubiaceae species density (SDwoody); (d) the ratio of herbaceous Rubiaceae species density to total Rubiaceae species density (Ratio). The blue line represents water and energy, the green line represents climate seasonality, the red line represents historical climate change, and the yellow line represents human influence. The environmental factors on the Y-axis include Annual Mean Temperature (AMT), Actual Evapotranspiration (AET), Temperature Seasonality (log-transformed) (TS-log), Precipitation Seasonality (log-transformed) (PS-log), AMT anomaly (AMTano), AP anomaly (APano), and Human Influence Index (HII), with * indicating significant effects.

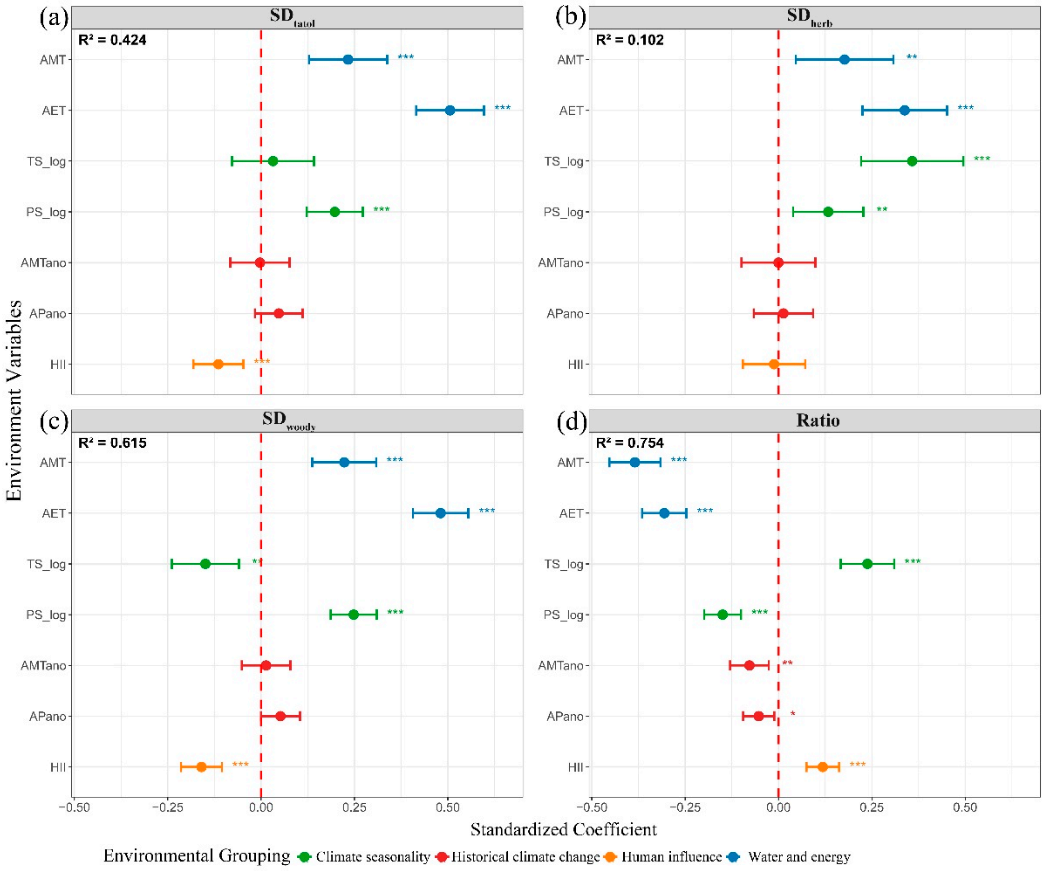

Fig. S5 Partitioning the variation in (a) Total Rubiaceae species density (SDtotal), (b) herbaceous Rubiaceae species density (SDherb), (c) woody Rubiaceae species density (SDwoody) and (d) the ratio of herbaceous Rubiaceae species density to total Rubiaceae species density (Ratio) among environmental variables in four groups (i.e. water and energy, the green line represents climate seasonality, the red line represents historical climate change, and the yellow line represents human influence variables).

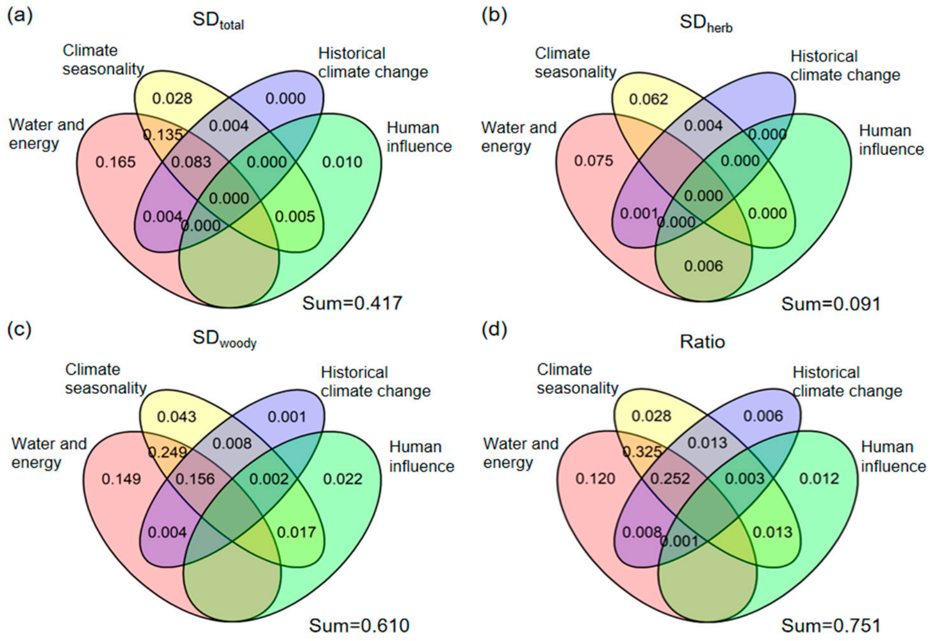

Supplement: Supplementary file 1 [file biology-14-01719-s001.zip › biology-3973543-supplementary/Supplement Figures.pdf]
